# A visual review: a study of gender power in Taiwanese cinema

**DOI:** 10.3389/fsoc.2026.1710446

**Published:** 2026-02-26

**Authors:** Yuting Yang, Ayu Haswida Abu Bakar, Weijie Zhao

**Affiliations:** 1 Film Program, Faculty of Film, Theatre and Animation, Universiti Teknologi MARA, Shah Alam, Malaysia; 2 Faculty of Built Environment, Universiti Teknologi MARA (UiTM), Seri Iskandar, Malaysia

**Keywords:** bubble charts, gender, power, Taiwan cinema, visual review

## Abstract

After the war and the gradual recovery of the economy and social construction, Taiwanese cinema also began to step into regular development. From the 1950s to the 2020s, Taiwanese cinema has undergone more than 70 years of development, from regional to international, and has become one of the major nameplates of Chinese-language cinema. As a country at the forefront of gender equality in Asia, Taiwanese films, regardless of period, have consistently paid special attention to gender. Following the turn of the new century, the portrayal of gender in Taiwanese cinema has become even more complex and diverse. However, academic research on Taiwanese cinema still focuses on Taiwan New Cinema and author films, and the attention paid to the study of gender in cinema is loose and unsystematic. This study aims to screen, analyse, and summarise gender power studies in Taiwanese cinema through an extensive literature search, focusing on the dynamics of gender power studies in Taiwanese cinema in the last five years (2020–2024). The analysis reveals that gender power studies in Taiwanese cinema are primarily based on director- or period-oriented film analyses, with intersectional studies being more prevalent, encompassing class, ethnicity, immigration, disability, politics, sexuality, and other related dimensions. Studies on gay and lesbian (queer)^1^ are the most numerous, followed by women, and finally, men. This study aims to present the review through bubble charts, utilizing visualization to provide a more graphic representation of the research dynamics of gender power in Taiwanese cinema. It will offer an overview of existing research issues, provide guiding suggestions for future research directions, and establish a research paradigm for film literature reviews.

## 1 Introduction

Since the 1950s, Taiwanese cinema has seen a gradual renaissance of the film industry fuelled by a boom in Taiwanese-language films, experiencing the revitalisation of Mandarin-language films, healthy realism films advocated by the government, accompanied by the popularity of Qiong Yao’s romantic melodramas, and interspersed with the short-lived emergence of genres such as social realist film and crime film, Taiwanese cinema has ushered in the epochal new wave of cinema-Taiwan New Cinema. After a brief period of splendour, Taiwanese cinema entered a trough. In 2008, *Cape No. 7* blew the trumpet for the revitalisation of Taiwanese cinema, and with the unfolding of commercialisation and genre incorporating indigeneity, Taiwanese cinema seemed to have found a marketable mode of development. Entering the 2020s, experiencing the impact of globalisation and neo-liberalism, and wrapped up in the post-epidemic and artificial intelligence era, Taiwan’s film industry is showing new development to varying degrees.

Gender-related issues have always occupied an important place in post-war Taiwanese cinema, with some films particularly featuring women as protagonists, and discussions on women’s issues are commonplace (Wang, 2020). Due to the profound influence of a patriarchal society, men dominate Taiwan’s society and culture, which in turn has led to insufficient discussion and attention to masculinity and male roles to circumvent the hindrance of male hegemony in progressive political space (Cho, 2024). In 2019, Taiwan legislated to endorse same-sex marriage (Kong, 2023). Taiwan’s gender affirmative action is at the forefront of Asia, which makes Taiwanese society more encompassing and open to queer people, and indirectly fuels attention to queer themes in the cultural field, presenting a very lively scene from visual presentation to academic discussion. Gender domination and resistance, gender oppression and liberation make gender a field of power (Duan, 2013, p. 6, my translation). Gender power involves individuals and groups of different genders, such as women, men, and gay and lesbian (queer). Especially in the new century, the portrayal of gender power in film has become more diverse and hybrid. However, the study of gender power in film has not been able to match the richness of the images. In particular, a notable gap exists in the study of gender power in cinema during the 2020s. Therefore, a literature review and analysis of the study of gender power in cinema can provide important references for the next direction of research.

Since the beginning of Taiwan New Cinema, Taiwanese films have received wider attention in the international market. In regional film studies, Taiwanese cinema is also a hotspot for research. It is again the Taiwan New Cinema that has received the most attention, with auteur film being widely discussed (Pickowicz and Zhang, 2020). Before the 1990s, a systematic compilation of gender representations in Taiwanese cinema according to a temporal spectrum focused mainly on female figures (Wang, 2020). With the rise and fall of Taiwan’s LGBT movement and the staged victory of marriage affirmation, attention to gay and lesbian (queer) themes has gradually climbed (Hsu, 2021; Sun, 2023; Tseng, 2024). Sex, age, class, and ethnicity have complex and contradictory entanglements with gender power (Duan, 2013, p. 67, my translation). Taiwan’s cinema has a long history of attention to class and ethnicity (Wang, 2020; Chao, 2020a; Fu, 2023; Hsieh, 2024; Tseng, 2024). As the impact of globalization began to increase the number of migrant workers in Taiwan, the intertwining of foreign immigrants and gender power began to be presented in images, which led to more discussion (Wang, 2020; Hsieh, 2024). There is also a part of the population that tends to be marginalized in real life and does not dominate in the presentation of images, so they are often overlooked. More attention should be paid to people with disabilities, especially the portrayal of their gender power in images (Tseng, 2024). These studies have adopted qualitative research methods. However, the existing research on gender power in Taiwanese cinema is still loose and unsystematic, and the research since the 2020s is even more blank.

This paper employs CASP (Critical Appraisal Skills Programme) Checklist: For Qualitative Research ensure trustworthiness (Critical Appraisal Skills Programme, 2024) and basic content analysis as a research method (Drisko and Maschi, 2016). Focusing on the period 2020–2024, this study examines five years of literature concerning gender power dynamics in Taiwanese cinema, with particular emphasis on women, men, and gay and lesbian (queer) gender groups within films. Relevant literature indicates that research on gender power concerns intersects with class, ethnicity, immigration, disability, and sexuality. The temporal scope of the research samples (based on film release dates) spans from the 1950s to the 2020s.

## 2 Method

Drisko and Maschi (2016, p. 13) suggest that “basic content analysis may be viewed as a hybrid research approach. [……], It is a research method that combines techniques from both research [qualitative and quantitative] traditions”. Employing the methodology of basic content analysis, this study involves meticulously reading and analysing literature relevant to the research objectives, followed by “coding.” Subsequently, the specific content within the resulting “*code names*” [the label allocated to *recording unit* (the term used by content analysts to identify text paragraphs or other materials bearing specific meaning), or text paragraph (Weber, 1990)] is examined [cited in Drisko and Maschi (2016, pp. 13, 41), retain the original italics], and the results of the coding and analysis are then visualised in the form of a bubble chart. We shall elaborate on this section through three phases: the theoretical framework, the problem and design, and the implementation process of the research.

### 2.1 Phase 1. *Theoretical framework*

This paper focuses on gender power studies in Taiwanese cinema. It centres on academic research concerning gender power within films, engaging in discussion and analysis. Here, it is necessary to elucidate the concept of “gender power” as it relates to the research. This study draws upon Chengli Duan’s research on gender politics, in which she contends that “the domination and resistance of gender, its oppression and liberation, render gender a field of power. The essence of gender politics lies in the power relations it encompasses. Gender politics primarily encompasses two dimensions: gender oppression and gender liberation. Yet it also involves the intricate entanglement of gender power relations with other power dynamics” (Duan, 2013, p. 6, my translation). Gender power reflects dominant and subordinate relationships between individuals or groups of different genders. Historically, gender power has manifested as inequality, with men typically occupying dominant positions while women and those who do not conform to gender norms are relegated to subordinate positions. Gender power frequently intersects with factors such as class, ethnicity, race, and others, giving rise to more complex power dynamics. Having clarified the theoretical framework, the subsequent discussion will address the research questions and research design.

### 2.2 Phase 2. *Questions and design*

The goal of this study is to review the current state of research on gender power in Taiwanese cinema over the past five years (2020–2024), which aspects of gender power this literature focuses on, what factors are of concern, and what eras are involved. With this in mind, we have identified the following research questions.

What factors are intertwined with gender power in studies about gender power in Taiwanese cinema?

What kind of interactive relationship is present between gender power and these factors in studies about gender power in Taiwanese cinema?

How does gender power relate to these factors in studies about gender power in Taiwanese cinema?

Based on the above research questions and in conjunction with the methodology of basic content analysis, the approach for conducting the literature search can be determined. Basic content analysis is primarily “deductive” in form. The researcher’s field of interest and preliminary coding are typically established before data collection and analysis, based on existing “theoretical” and empirical research (Drisko and Maschi, 2016, pp. 21, 22). The scope of the research was delineated by searching digital databases using specific sets of keywords. Then, “a thorough manual search” was conducted by individually browsing the relevant journals and book chapters. This “dual strategy” ensured the comprehensiveness of the search scope, thus including relevant research papers in the field of humanities and social sciences in the search (Zhang et al., 2024, p. 16867).

“Researchers use *descriptive designs* [a method for basic content analysis research design] to provide information that details the character and quality of a sample or population” [Anastas, 1999, cited in Drisko and Maschi (2016: 33), retain the original italics]. We used four mainstream digital databases related to the humanities and social sciences: the Airiti Library華藝線上圖書館, Scopus, Web of Science, and Jstor. Here we have included a Chinese language academic database based in the Taiwan region, which contains a wealth of resources for scholarly research in the Taiwan region (Airiti, 2025). This is because the subject of my research is Taiwanese cinema. The search keywords were derived from the research questions, which were: Taiwan, Taiwanese, cinema, film, gender (including women, men, gay, lesbian, queer), and power; 臺灣電影, 性別, 權力 (traditional Chinese, Airiti Library search keywords).

Inclusion and exclusion criteria were needed to ensure more accurate search results (Zhang et al., 2024, p. 16867). In the inclusion criteria, only journal articles and book chapters written in English or traditional Chinese were included. The publication period was the last five years, from 2020 to 2024. In the exclusion criteria, materials that did not contain the search terms were excluded, and articles were excluded if they did not include Taiwan/Taiwanese cinema/film and gender (including women, men, gay, lesbian, queer) in the article title (considering that some of the book chapters did not have abstracts) or in the abstract.

At the same time, the quality of each article was assessed using the Critical Appraisal Skills Programme (CASP) checklist. “Critical Appraisal is the process of carefully and systematically examining research to judge its trustworthiness, and its value and relevance in a particular context. [……], Critical appraisal skills are important as they enable you to assess systematically the trustworthiness, relevance and results of published papers.” Critical appraisal can be applied to qualitative research. “An in-depth analysis of a phenomenon based on unstructured data, such as interviews, observations, or written material. It’s often used to gain insights into behaviours, value systems, attitudes, motivations, or culture” (CASP, n.d.). The implementation phase was started based on research questions, search keywords, inclusion criteria, exclusion criteria, and quality assessment.

### 2.3 Phase 3. *Implementation process*

**Figure 1:**
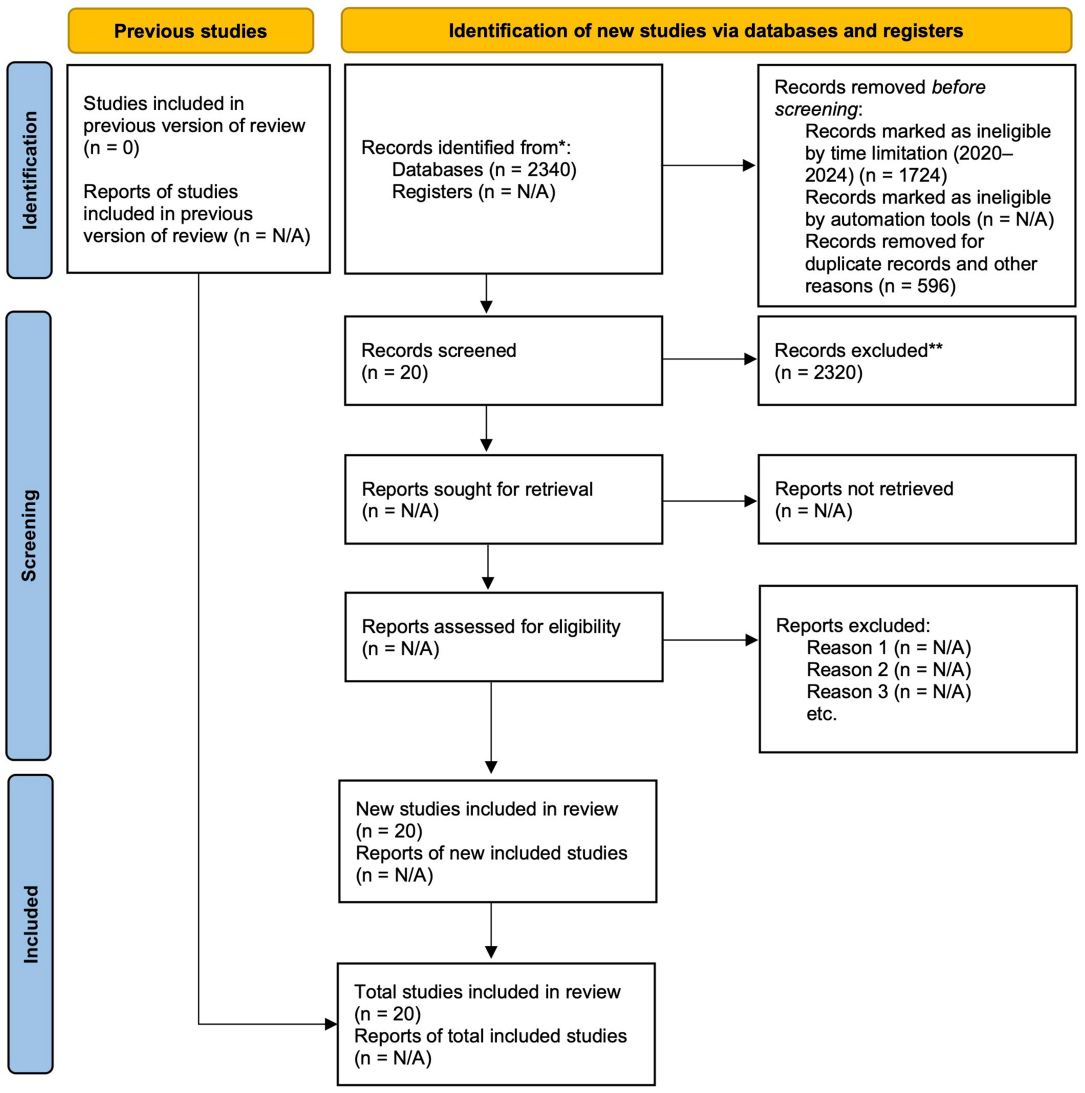
PRISMA 2020 flow diagram for updated systematic reviews which included searches of databases and registers only (Page et al., 2021).

“Sampling in content analysis is often multistage in nature. [……]. Such multistage sampling techniques seek to identify transparently the most relevant data to address the research question” (Drisko and Maschi, 2016, pp. 36, 39). A *sampling frame* (p. 37, retain the original italics) is determined by employing multistage sampling techniques. A comprehensive search was conducted on four different digital platforms based on the search terms identified by the research questions described in the previous section. Initially, 2,340 articles were retrieved. Next, restricting the time to the last 5 years from 2020 to 2024, a further 616 articles were retrieved. A careful manual search, removing duplicate and unrelated articles and selecting only journal and book chapters, retrieved 37. Then, based on the abstracts and keywords of the articles and consideration of the exclusion criteria, 21 papers were retrieved that fulfilled the criteria. Finally, the papers were assessed for quality, and only 20 articles (sampling frame) were fully relevant to the review theme and objectives. The Prisma 2020 flow diagram (Figure 1) illustrates the screening process for research samples. “All content analysis is a form of data reduction. [……]. Massive amounts of data can be succinctly summarized using content analysis” (Drisko and Maschi, 2016, p. 34). Next, we shall analyse and summarise the content of the relevant research papers.

## 3 Results and discussion

Through a careful review of the selected papers, it was determined that they were primarily concerned with three dimensions of gender power: men, women, and gay and lesbian (queer), covering the years from the 1950s to the 2020s, with an intersection of individual code names of concern for each dimension. Men focused on six code names, women focused on twelve code names, and gay and lesbian (queer) focused on eighteen code names. We will further elaborate on the code names that each dimension focuses on and explain how they relate to gender power below.

### 3.1 Phase 1. *Men*

The Men dimension concerns nationalism, identity, masculinity (frustrated and lost, Place-based/*Beiqing*, hegemonic), and impotent men.

#### 3.1.1 Nationalism

Through textual analysis of the film *The First Error Step* (aka, *Never Too Late to Repent*, 錯誤的第一步, dir. Tsai Yang-Ming, 1979). Ting-Wu Cho argues that this social realist film work is full of “nationalist imagination” and that the male protagonist’s story exemplifies the legacy of Japanese colonial rule, the authoritarian politics of the Kuomintang and the islanders’ history of nativism intertwined in a multilayered power relationship of debate and negotiation that Taiwan experienced during the Cold War (Cho, 2024, pp. 257, 271). Yun-Liang Kan and Chia-Jung Tsai provide a discourse analysis of masculinity in the film *Kano* (dir. Umin Boya, 2014). They argue that sports films help to construct and imagine a national consciousness of the community (Kan and Tsai, 2022, p. 423). Typical masculinity is closely related to nationalism [Parry et al., 2021, cited in Kan and Tsai (2022, pp. 423–424), my translation].

#### 3.1.2 Identity

Mei-Hsuan Chiang’s analysis focused on director Tsai Yang-Ming’s gangster trilogy *Gangland Odyssey* (aka, 大頭仔, dir. Tsai Yang-Ming, 1988), *Fraternity* (aka, 兄弟珍重, dir. Tsai Yang-Ming, 1990), and *Joe-Goody* (aka, 阿呆, dir. Tsai Yang-Ming, 1992). She argues that the gangster protagonists in the three works have a crisis of identity construction because they are unable to gain the approval of their biological fathers or become approved fathers. This crisis of the father-son relationship responds to the identity dilemma that arose under the rapid development of Taiwanese society from the late 1980s to the early 1990s (Chiang, 2022, pp. 24, 33, 35, 36).

#### 3.1.3 Masculinity

Frustrated and lost masculinity: Mei-Hsuan Chiang’s study of Taiwanese gangster films from the late 1980s to the early 1990s focuses on director Tsai Yang-Ming’s gangster trilogy from this period, arguing that the portrayal of gangsters in the films presents frustrated and lost male masculinity (Chiang, 2022, p. 44). In another of her articles examining women’s roles in Taiwanese gangster crime films, Mei-Hsuan Chiang argues that “in recent studies on Taiwanese gangster films after the post-martial law, several scholars have observed the frustrated and lost masculinity presented by Taiwanese gangster films during this period” (Liao, 2015; Chiang, 2024, p. 27, my translation, some of the words used are from the original).

Place-based masculinity/*Beiqing* masculinity: In the social realist film *The First Error Step*, Ting-Wu Cho argues that the film reinvents “place-based masculinity”, which is rooted in the traditional melancholic tone of Taiwanese-language cinema [Zhang, 2013, cited in Wang (2020, p. 303)], and defines it as “*beiqing*” masculinity (Cho, 2024, p. 257). This “beiqing masculine” emphasises the “woeful fate” of the male protagonist, the “effeminization of men”, and the violent reproduction of colonial masculinity [Shie, 2009, cited in Cho (2024, p. 265)]. This reproduction and intensification of violence are reflected in the male-dominated underworld of the film (Cho, 2024, pp. 260, 266–267).

Hegemonic masculinity: In the late 1970s, woman’s revenge films began to appear, and until the mid-1980s, the genre presented an unassailable position of hegemonic male consciousness. Since the 2000s, gangster films have reinforced the genre’s masculine traditions with gang fights and brotherhood (Chiang, 2024, pp. 25–27, 29, my translation). Yun-Liang Kan and Chia-Jung Tsai argue that sports films are a set of scripts that celebrate masculinity by analysing the film *Kano* [Walton-Fisette et al., 2017, cited in Kan and Tsai (2022, p. 416), my translation]. Sports films are categorised as the main genre for displaying masculine qualities [Chao, 2024, cited in Kan and Tsai (2022, p. 427), my translation].

#### 3.1.4 Impotent men

In local post-war Taiwanese literature and cinema, impotent men are mainly represented in two types: those who grow up without the support of a good family or live in an environment where a strong father figure is absent tend to become typical impotent men, who are economically or socially dependent on women; and the other is the irresponsible vagabonds, a street hooligan without a steady job (Cho, 2024, p. 264). Chun-Chi Wang mentions that male protagonists in Taiwanese film melodramas often have insufficient capacity and cites Shen Shiao-Ying’s study that male protagonists in Taiwanese-language film melodramas are usually impotent [Shen, 2017, cited in Wang (2020, p. 303)]. Evelyn Shih, in her discussion of 1960s Taiwanese-language spy films, argues that male spies ultimately prove to be incompetent in films centred on female spies (Shih, 2020, p. 115).

Research on men’s gender power in Taiwanese cinema reveals that most studies focus on masculinity, with the chosen film study samples spanning the 1970s to the early 1980s and the late 1980s to the early 1990s. The most attention was paid to gangster films, especially the works of director Tsai Yang-Ming. Although men dominate, diverse masculinities are mentioned more often than a single hegemonic masculinity, especially as these masculinities are rooted in place and have a distinctive indigeneity.

### 3.2 Phase 2. *Women*

Women’s dimension concerns national allegories/sentiment, ethnicity, stereotype (femininity, femme fatale), patriarchal, sexuality, agency and subjectivity, religion, male gaze, marginal status, class, rape culture (sexual and gender violence), and identity.

#### 3.2.1 National allegories/sentiment

Chun-Chi Wang suggests that representations of women and gender relations in Taiwanese cinema are often “nationally embedded.” After the restoration of Taiwan in the mid-1950s, Taiwanese-language films began to develop, with melodrama and spy films being the two genres of Taiwanese-language cinema, both of which feature female protagonists who dominate the story and drive the plot forward. Gender is often used as a metaphor in Taiwanese-language melodramas to express the various emotions of Taiwanese society during the transition period. Taiwanese-language spy films replace male spies with female spies, and these female heroes are seen as symbolic repositories of “national sentiment” (Wang, 2020, pp. 302, 303, 304). In Evelyn Shih’s discussion of two 1960s Taiwanese-language spy films, she argues that the more marginalised position of the female spy in the films better carries the historical position of the post-war Taiwanese film audience. The empowerment of women and the empowerment of historical positions are closely connected through the 1960s Taiwanese-language female spy films (Shih, 2020, pp. 123, 124). In the 1960s, a temporary parallel to Taiwanese-language cinema was the government’s promotion of healthy realism, with female-centred private stories, which could be described as “*national allegories*,” with women acting as the narrative arena on which “*nationalist aspirations*” could be projected (Wang, 2020, pp. 304, 305).

#### 3.2.2 Ethnicity

Taiwan’s aboriginal groups are often marginalised and receive little attention. The 1980s saw the rise of aboriginal power discourse; however, they have long been in a situation where they are both stigmatized and romanticized by the media (Wang, 2020, p. 308). In media representations, Aboriginal people are not only subjected to stereotypical impressions, however are also constantly subjected to Sinicised and patriarchal heteronormativity. In particular, aboriginal women are often doubly exploited in such situations; the first level of exploitation is their otherness in the tribe, with the responsibility for cultural transmission remaining with young men (Wang, 2020, p. 310). Another layer of exploitation is their position as sinners in the revitalisation and prosperity of the ethnicity, seen as the culprits of Han modernity. Because of their reproductive nature, they are often marginalised as “single lower-class male mainlanders” seeking marriage [Wang, 1993, cited in Wang (2020, p. 309)]. In the process of intermarriage with the Han, they are disciplined and taught by Han culture, which also means “the obliteration of indigeneity” (Wang, 2020, p. 309). Chun-Chi Wang has another perspective on aboriginal women choosing to be more connected to the outside world and leaving their tribes. She cites the film *Finding Sayun* (aka, 不一樣的月光, dir. Laha Mebow, 2011) as an example of “a provocative reminder of the implicit gender oppression in the women’s own tribe” (Wang, 2020, p. 310). In films about Taiwan’s aborigines, gender oppression and advancement are often associated with ethnicity (Wang, 2020, p. 310). Yu-wen Fu discusses “women’s narratives” in the Taiwanese film *Seediq Bale* (aka, 賽德克·巴萊, dir. Wei Te-Sheng, 2011), arguing that the film gives a voice to “doubly silenced women” through effective cinematic language. Women’s “the gendered, interventional narratives”, on another level, help people to understand “Austronesian Indigenous cultural cores” and “Indigenous identity”. The stories of women in the film reflect “the connectedness of gender and ethnicity”, with women taking on the difficult task of national “bloodline and cultural identity” (Fu, 2023, pp. 158, 161, 168, 169). Hsin-Chin Hsieh uses two short films about migrant workers as an example to analyse the presentation of migrant workers’ gender power in a foreign country. In the analyse short film, *Tea Land* (aka, 高山上的茶園, dir. Tseng Ying-Ting, 2018), argues that “the female body becomes a battleground for men of different ethnicities, remaining in the power cage of gender, ethnicity and the state” (Hsieh, 2024, p. 65, my translation).

#### 3.2.3 Stereotype (femininity, femme fatale)

Xuelin Zhou and Chun-Chi Wang both mention that although the Qiong Yao films of the 1970s and 1980s showed women’s roles differently from those of the 1960s, reflecting an increased sense of female autonomy, there was still a traditional aspect to the portrayal of women. “The conventional view of female beauty still emphasises their physical attractiveness to men, [……], they still embody contradictions as both the subject and the object of the gaze” (Zhou, 2024, p. 101). “Femininity for women is often infused with emotionality” (Wang, 2020, p. 306). At the end of the 1970s, woman’s revenge films began appearing under the social realist films craze. Take Tsai Yang-ming’s famous woman’s revenge film *Woman Revenger* (aka, 女性的復仇, dir. Tsai Yang-ming, 1981) for example. The film seems to show female-led vengeance; however, the display of women’s naked bodies and the ending’s punishment of the women show that women are still disciplined in the ‘male-dominated gender ideology’ (Chiang, 2024, pp. 25, 26, 27, my translation). Chun-Chi Wang offers another perspective, arguing that the ambivalence presented in the film can be interpreted as “a reaction and backlash to the changing role of women and bourgeoning feminism since the late 1970s” (Wang, 2020, p. 307). Focusing on the film *Joe-Goody*, Mei-Hsuan Chiang argues that the role of Sha Li, the gangster’s sister-in-law, “resembles the femme fatale common in the film noir” (Chiang, 2024, p. 29, my translation, some of the words used are from the original).

Stereotypes are also reflected in the presentation of female migrant marriages, and Chun-Chi Wang suggests that the mainstream media often portrays female marriage migrants in negative stereotypes. Prejudice against backward countries is transferred to these migrants, and marriage migrants are often involved in monetary transactions. Therefore, female marriage migrants are often stereotyped as women who are profit-oriented and degenerate (Wang, 2020, pp. 311, 312). However, in an attempt to reverse the stereotypical image of female marital immigrants, some Taiwanese films show positive images of women with good qualities, such as loyalty. These women are often silent and do not have much autonomy in their families. While such portrayals refute the stigmatisation of immigrant women and strongly respond to stereotypes about them, they make the characterisation one-dimensional, and “run the risk of sacrificing diversity” (Wang, 2020, p. 312).

#### 3.2.4 Patriarchal

Xuelin Zhou discusses the Qiong Yao films produced by the Grand Motion Picture Company (GMP) in the 1960s. Qiong Yao’s films of this period were influenced by the context of the times, “the Confucian-oriented ideology toward women dominated.” The films present the “gender-based/biased authoritarian hierarchy” was closely related to “the patriarchal social structure and the mainstream political ideology (Zhou, 2023, p. 367). In another article by Xuelin Zhou examining Qiong Yao films in the late 1970s and 1980s, he argues that in the film *The Wild Goose on the Wing* (aka, 雁兒在林梢, dir. Liu Lili, 1978), the heroine consciously uses “femininity and sexuality” as a means of challenging “patriarchal power,” attracting men and then “destroying” them (Zhou, 2024, p. 100). Discussing the social realist rape-revenge films of the late 1970s and early 1980s, Chun-Chi Wang argues that these films share common characteristics, citing Pam Cook’s theory that patriarchal ideas are still deeply rooted and that although women can determine their destiny, character development is based on the idea of putting “the woman in the man’s place” [Cook, 1976, cited in Wang (2020, p. 306)]. Mei-Hsuan Chiang argues that “female revenge narratives ultimately fail to challenge patriarchy” in early 1980s woman’s revenge films (Chiang, 2024, p. 29, my translation). Hsin-Chin Hsieh analyses a short film, *Merah* (aka, 紅色, dir. Liu Chun-Yu, 2020), which concerns unintended pregnancies among female Indonesian migrant workers. She suggests that “female migrant workers’ bodily and reproductive rights are subject to multiple patriarchal constraints by employers, intermediaries, and the state” (Hsieh, 2024, p. 73, my translation). Tingting Hu discusses female “active perpetrators” in Chinese cinema, focusing on Taiwanese films *The Bold, the Corrupt and the Beautiful* (aka, 血觀音, dir. Yang Ya-che, 2017). In her article, she focuses on Mrs. Tang, one of the female protagonists, and argues that female violence and transgression are a form of “dramatised and visible phallic power,*”* which women use to seemingly challenge male dominance and gain gender equality. However, in essence, “the patriarchal authority, as the powerful, dominant side,” what women are allowed to have is power within a specific range of “controllable and non-threatening.” Once a woman’s transgressive behaviour exceeds this range, it needs to be eliminated (Hu, 2021a: 86). In another article in which the author participated, they discuss the same film from a “postfeminist” perspective and shared the same viewpoint. They aruge that the seemingly powerful female figures in the film are presented in a “stigmatic” manner, attempting to equate female power with negativity, thereby fundamentally undermining feminism (Hu and Guan, 2021b, p. 1689). Jiaying Sim, in discussing Film *Nina Wu* (aka, 灼人秘密, dir. Midi Z, 2019) with sexual and gender violence, pointed out that “Nina Wu problematises rape culture as a symptom of deep-seated misogynist and patriarchal structures of power that protect perpetrators within the Taiwanese film industry” (Sim, 2023, p. 206).

#### 3.2.5 Sexuality

During the Taiwan New Cinema period, Edward Yang directed *Expectation*, which was included in the omnibus film *In Our Time* (aka, 光陰的故事, dir. Tao Te-chen, Edward Yang, Ko I-chen, Yi Chang, 1982, marking the launch of Taiwan New Cinema), a film that explicitly depicted female sexuality and boldly and bluntly eroticised the male body through female viewing. Several films adapted from novels about women’s issues, depicted by women writers, presented women living in the grips of patriarchy and struggling with their sexuality. Until the 1990s, with the rise of women as a whole and the general advancement of modernization, partly contributed to the emergence of films dealing with issues of traditional gender norms, particularly heterosexual love and marriage based on sexuality (Wang, 2020, pp. 307, 308). Hsin-Chin Hsieh’s article focuses on two short films about migrant workers, discussing the sexuality and bodily autonomy of migrant workers from different countries in Taiwan. Focusing on the private emotions and sexuality of migrant workers, she explores the complexity of migrant workers’ sexual politics. Sexuality has become an important part of migrant workers’ human rights narratives (Hsieh, 2024, pp. 55, 73, my translation).

#### 3.2.6 Agency and subjectivity

Xuelin Zhou discusses the female protagonists in Qiong Yao films made by director Liu Lili in the late 1970s and early 1980s. Unlike the Qiong Yao films produced by GMP, a company founded by Li Han-xiang in the 1960s, the female protagonists in these films display “independent agency and subjectivity” in their relationships with men (Zhou, 2024, p. 94). Chun-Chi Wang argues that some Qiong Yao films rely heavily on the general conventions of “gothic literature.” She cites two Qiong Yao films from 1971 and 1980 as examples of how a woman’s “affective presence” completely influences the men around her, who are expected to be responsive and even subservient to her needs (Wang, 2020, p. 306). In her discussion of atypical female roles in Taiwanese gangster crime films since the 1980s, Mei-Hsuan Chiang suggests that there are several atypical cases in a handful of gangster films from the 1980s to the 1990s that “construct the position of the female core in the gangster genre narrative.” Director Tsai Yang-ming’s late 1980s gangster films, in which the role of the gangster’s female companion still occupies a significant part, “subverted the gangster genre’s feminine imagery of women.” The dominant female companion role in gangster films of the late 1980s and early 1990s “loosens the familial and male-dominated narratives at the heart of the genre, highlighting female agency and instead threatening patriarchal structures” (Chiang, 2024, pp. 25, 27, 29, my translation).

#### 3.2.7 Religion

Hsin-Chin Hsieh discusses the short drama film *Merah*, suggesting that the female protagonist’s bodily autonomy and reproductive rights are in the midst of a multi-powered tug-of-war, and that for migrant workers of faith, religion becomes a part of the multi-poweredness and is related to their bodily autonomy (Hsieh, 2024, p. 72).

#### 3.2.8 Male gaze

Chun-Chi Wang, in her discussion of gender representations in genre films of the 1970s and 1980s, notes that in the late 1970s and early 1980s, films with women at the centre of the narrative were as popular as social realist films and Qiong Yao films. Among them, rape-revenge films dominated. “The position of female protagonists aligns with Laura Mulvey’s male gaze theory” [Mulvey, 2004, cited in Wang (2020, p. 306)]. Mei-Hsuan Chiang makes a similar point in discussing atypical female characters in Taiwanese gangster crime films since the 1980s. Taking the woman’s revenge films of the early 1980s as an example, the presentation of the naked female body became “an object of erotic fantasy for the male audience on the other side of the screen, just like the object of desire under the male gaze as discussed by feminist film scholar Laura Mulvey” [Mulvey, 1975, cited in Chiang (2024, 26, my translation)].

#### 3.2.9 Marginal status

Chun-Chi Wang argues that in Taiwanese films, the suffering of the female protagonist symbolises the oppression of capitalism. Still, this metaphorical use of women’s plight indirectly affirms women’s marginal position in society (Wang, 2020, p. 303). Mei-Hsuan Chiang analyses atypical female roles in Taiwanese gangster crime films and argues that “since 2000, the roles played by women in gang-themed films have once again been marginalised” (Chiang, 2024, p. 29, my translation).

#### 3.2.10 Class

Chun-Chi Wang argues that gender and class are interrelated axes prevalent in Taiwanese-language film melodramas. The saviour characters in Taiwanese-language melodramas tend to be of a higher social class, regardless of gender. Qiong Yao films also portray many women from different social classes; however, class is not always a significant factor in triggering love rivalries. Until the 1990s, the “social division” most intertwined with gender was class; however, class was not positioned as an important factor influencing women’s roles in marriage and family (Wang, 2020, pp. 303, 306, 308). In Hsin-Chin Hsieh’s article exploring the sexuality and sexual politics of immigrant workers, she suggests that there is a class difference between immigrant workers and employers, that immigrant workers are at the bottom of the social ladder, and that basic needs and rights are ignored (Hsieh, 2024, pp. 57, 68). Chun-Chi Wang also claims the issue of “class-inflicted pain” and builds on broker-mediated transnational marriage in her discussion of women’s rights in transnational marriage (Wang, 2020, p. 312).

#### 3.2.11 Rape culture (sexual and gender violence)

Through the film *Nina Wu*, Jiaying Sim discusses the problematic presentation of sexual and gender-based violence in the media industry focuses on how rape culture is realised in the Taiwanese film and media industry. Rape culture socially, culturally, and politically condones and rationalises violence against women, and places the blame for violence against women victims (and other victim-survivors). [Buchwald et al., 2005; Fileborn and Loney-Howes, 2019, cited in Sim (2023, pp. 204, 217)].

#### 3.2.12 Identity

Beth Tsai explores the exploitation of women by the film industry through “soundscape,” camera shots, and other cinematic audiovisual analyses of the film *Nina Wu*. She argues that the film is an exploration of women’s search for “identity and self-affirmation” after suffering trauma in the workplace. At the same time, extending the larger theme, in the context of globalisation, Midi Z’s work, whether it is about migration, economics, or gender, is hindered by “the larger political and capitalist forces” (Tsai, 2023, pp. 119, 126, 127).

Under the women’s dimension, it can be seen more clearly that *patriarchal* is the one that receives the most attention; however, the years involved are relatively concentrated in the 1950s to the early 1980s, and after the 2010s, discussions about the gender power of immigrants are once again concerned with patriarchy. The *class* has appeared in almost every stage of Taiwan’s film development. Although its intersectionality with women’s gender power is sometimes less important, it has almost become one of the necessary elements in film narratives. With the advent of globalisation, the topic of immigration has gained prominence. The film’s focus on migration extends the breadth of gender issues. It is not difficult to find that immigration leads to more complex issues, which are related to race, class, nation, religion, gender, and so on, and whether it is immigrant marriage or immigrant workers, what kind of gender, all of them are at the bottom and the edge of the power structure, and should receive more attention and concern.

From the period of Taiwanese-language films to contemporary films, it is not difficult to find that the presentation of women’s gender power in films is closely related to the feminist movement in Taiwan. Discussions concerning women have gradually evolved from grand themes to personal subjects and from symbolic representations to individual concerns. However, it has to be admitted that the discussion of women’s gender power reflects a pessimistic attitude, the problems of women’s rights and interests caused by power inequality are still numerous and complex, and the task of striving for gender equality is still arduous, especially concerning the rights of ethnic minority women, immigrant women, and sexual and gender violence, which is even more marginalised.

### 3.3 Phase 3. *Gay and lesbian (queer)*

Gay and lesbian (queer) dimensions concern identity, ethnicity, class, indigenous, sexuality, Tongzhi^2^ camp, disability, heterosexual patriarchy, narrative homonormativity, same-sex marriage, come out of the closet, de-stereotyping, family, diverse family formation/gender-diverse family, gender performativity, familial performativity, queer sphere, queer gaze.

#### 3.3.1 Identity

Shi-Yan Chao argues that while the film *Outcasts* (aka, 孽子, dir. Yu Kan-ping, 1986) is “incorporating” the local, the film further highlights one of the shortcomings of the local discourse, which tends to ignore “gay identities and queer issues,” that is to say, is “characteristically heteronormative” (Chao, 2020a, p. 60). Director Zero Chou said that for her, ‘*tongzhi* is an aesthetic as well as an identity.’ Shi-Yan Chao argues that Zero Chou’s work shows that “sharply contradict” no exists between “*tongzhi* identity” and Taiwanese culture (Chao, 2020b, p. 230, retain the original italics). Hsiu-Ping Tseng discusses *Drifting Flowers* (aka, 漂浪青春, dir. Zero Chou, 2008), argues that “the *po* [‘*tomboys* partners are often normatively-feminine women who are labeled *po* (wife)’ (Fung, 2021, p. 141)], influenced by heteronormative stereotypes, are often overlooked for their queer identities and the ‘subjectivity of *po’* in the film.” On the other hand, in “lesbian theme cinema, there are few images of the masculine *T* [among lesbian communities in Hong Kong, Taiwan and mainland China, ‘a group of masculine-presenting, assigned-female-at-birth individuals have come to be known as *tomboys*’ (Fung, 2021, p. 141)], and there have even been critical voices of *T* and *po* culture” (Tseng, 2024, pp. 46, 50, my translation, some terms are derived from the original work).

#### 3.3.2 Ethnicity

Shi-Yan Chao argues that “one crucial intervention in this Chinese queer diasporic imaginary^3^ is the rethinking of ethnicity on the island of Taiwan.” Since the 1990s, with the rise of Taiwanese consciousness and the vigorous development of multiculturalism, films focusing on ethnic minority queers have begun to proliferate. Shi-Yan Chao cites the film *Tale of the Lost Boys* (他和他的心旅程, dir. Joselito Altarejos, 2017) as an example of a film that reflected “the double marginalization of aboriginal by heteronormative Han Chinese culture” (Chao, 2020a, pp. 95, 96).

#### 3.3.3 Class

Hsiu-Ping Tseng discusses the film *Drifting Flowers*, arguing that “class and gender (especially the economic pressures faced by lesbians, the dilemmas of patriarchal structures) are the focal points reproduced in the film” (Tseng, 2024, p. 42, my translation).

#### 3.3.4 Indigenous

Shi-Yan Chao, discussing the film *Outcasts*, argues that the film challenges “Chinese familialist nationalism” on two levels, one of which is the focus on “the native and the local.” At the same time, the character of the film contributes to the distinctive expression “queer diaspora.” This expression stands in opposition to the often unchallenged prevalence of the “queer culture” paradigm centred on the West, especially in the United States of America (Chao, 2020a, pp. 60, 61). In another article by Shi-Yan Chao discussing the aesthetic of *Tongzhi* camp, he adopts a case study approach to discuss Zero Chou’s film, and in analysing the film *Splendid Afloat* (aka, 豔光四射歌舞團, dir. Zero Chou, 2004), he argues that the “camp aesthetic” of the film strategically adopts a “peculiar indigenous manifestation.” Through the film, the director proved that “*tongzhi* culture, [……], is firmly ingrained in this island [Taiwan]” (Chao, 2020b, pp. 219, 230, retain the original italics). In Taiwan’s gay/lesbian youth films from 2002 to 2011, some film creators were themselves tongzhi and took a more queer approach to responding to the heteronormative censorship mechanism. Zu-Xin Sun argues that “this tactic was aggressive and offensive, and thus in the process of the narrative, the directors focused more on indigenous experiences, with the recreation of collective memories to gain the emotional connection and resonance of the local” (Sun, 2023, pp. 42, 43, my translation).

#### 3.3.5 Sexuality

Hsiu-Ping Tseng, in discussing the film *Drifting Flowers*, lesbian disability sexuality, contends, “often the sexuality of non-heterosexual female disability is doubly discriminated against and easily hidden in the ideology of able-bodied, invisible and unseen, becoming micro-discrimination that is seen everywhere in life” (Tseng, 2024, p. 46, my translation). Nicholas de Villiers, in the book *Cruisy, Sleepy, Melancholy: Sexual Disorientation in the Films of Tsai Ming-liang* (2022), reevaluated Tsai Ming-liang’s major works to propose a new theory on “relationships among queer sexuality, space, and our experience of cinema” (De Villiers, 2022).

#### 3.3.6 Tongzhi camp

Chun-Chi Wang argues that one way to address “*gender deviance*” in tongzhi films is through the use of “*camp*.” She cites Katrin Horn’s research to explain the “*camp*,” a term that draws on scholarship from the 1990s, “an excessively stylized parody and in-group humor capable of intervening in naturalised and naturalising discourse while granting access to otherwise oppressive systems of meaning-and pleasure-making” [Horn, 2017, cited in Wang (2020, p. 313)]. Shi-Yan Chao first proposes that “gay camp” is a specific camp discourse typified by Newton and Babuscio’s views, and give a definition of camp that “camp features irony/incongruity, theatricality, aestheticism/stylization, and humor, and all these features are of peculiar relevance to homosexual experience.” Next, he further points out that in the context of Chinese culture, dissecting the relationship between the concept of “gay sensibility” (which is supplemented by an emotional structure mediated by “gay shame and gay melancholy,” both of which carry special Chinese connotations) and social oppression was the basis for him to translate “gay camp” as “*tongzhi* camp.” Tongzhi camp agents continue to negotiate with the negative emotions driven by tongzhi’s experiences of marginalisation, typified by “gay shame and gay melancholy,” and despite the social and historical terms changing, this situation of marginalisation continues to haunt “*tongzhi* subjects” in mainstream Chinese society (Chao, 2020b, pp. 206, 207, 244, retain the original italics).

#### 3.3.7 Disability

Hsiu-Ping Tseng believes that the film *Drifting Flowers* “highlights the multiple disadvantaged situations of a visually impaired lesbian woman who is also disabled, female, and a lesbian. [……], such an inclination to ignore research on people with disabilities has persisted in Taiwan’s film studies for a long time” (Tseng, 2024, p. 43, my translation).

#### 3.3.8 Heterosexual patriarchy

After analysis, Tseng Hsiu-ping opines: “concepts of marriage and family in the context of Han Chinese kinship structure and ideology, lesbians endure oppression from both patriarchy and heterosexual hegemony in terms of both gender and sexuality.” In the film *Drifting Flowers*, the portrayal of the story of an elderly lesbian demonstrates her long-standing “queer trauma,” which is “the shadow left by heteronormative hegemony and the father” (Tseng, 2024, pp. 44, 57, 58, my translation).

#### 3.3.9 Narrative homonormativity

When Chun-Chi Wang discusses the film *Formula 17* (aka, 17歲的天空, dir. Chen Yin-Jung, 2004), she points out that the film’s portrayal of the masculine appearance of the two male protagonists made them look “natural” like ordinary heterosexual men. This implicit meaning of “genderly normalising” tongzhi characters is a compromise to the heterosexual society, making certain types of tongzhi individuals more acceptable than those who violate the binary gender system. This is also a characteristic of Taiwanese tongzhi films, which “by and large, comply with conventional narrative norms.” This helps to increase society’s acceptance of *tongzhi* (Wang, 2020, pp. 313, 314, 312). Zu-Xin Sun holds that *“*queer themes in Taiwan during the 1990s were incorporated into traditional Eastern culture. In terms of themes, choosing homosexuality as the focal point was queer and marginal; however, in terms of narrative strategies, it was moderate and reconciliatory. This became a characteristic of queer practice in Taiwanese gay/lesbian films. [……]. From 2002 to 2011, during this period, Taiwanese gay/lesbian films mainly presented homosexual images to the mainstream society through an Eastern reconciliatory approach, constructing a youthful and sunny image of homosexuality that conformed to multiple traditional moral norms” (Sun, 2023, pp. 38, 42, my translation, some terms are derived from the original work). When Ju-Ting Hsu was researches the queer imagery of Taiwan’s new generation female directors, she proposes that “the narrative of the new generation female queer imagery directors constructs a set of cultural logic of homonormativity of homosexuality/queerness, that is, it simulates a queer practice landscape where homosexuality and heterosexuality are the same and contrasted with each other, and through the narrative homonormativity, it subverts the hegemonic discourse of mainstream heterosexuality.” (Hsu, 2021, p. 77, my translation, some terms are derived from the original work).

#### 3.3.10 Same-sex marriage

Yenna Wu suggests that the film *Your Name Engraved Herein* (刻在你心底的名字, dir. Patrick Kuang-Hui Liu, 2020) is a “timely cultural production” that helps bridge the gap still existing between the official legislation and public perception regarding “same-sex marriage” (Wu, 2021, p. 73). Zu-Xin Sun argues that queer films in Taiwan from 2012 to 2020 began to explore “diverse family formations imagined” in “the era of marriage equality. [……], Queer films of the era of ‘marriage equality’ have moved beyond the gay/lesbian film genre of the previous decade and continue to explore various possibilities that queer can achieve in the field of family and marriage” (Sun, 2023, pp. 43, 45, my translation, some terms are derived from the original work).

#### 3.3.11 Come out of the closet

In discussing the queer changes in Taiwanese cinema of the new century, Zu-Xin Sun proposes: “In the early 21st century, a series of works represented by Blue Gate Crossing (藍色大門, dir. Yee Chih-yen, 2002) created the ‘sub-genre of Taiwanese gay/lesbian youth films.’ Against the backdrop of the pressure of the ‘coming out’ in the LGBT movement, these sub-genre films achieved ‘visual coming out’ of homosexuals, [……], and also conveyed the anxiety of coming out to the public through images.*”* The author further argues that *“*the concentrated explosion of gay/lesbian films between 2002 and 2011 practiced a roundabout strategy that could be called ‘visual coming out.’ [……], it becomes the narrative feature of Taiwanese gay/lesbian youth films during this period” (Sun, 2023, pp. 35, 40, 42, my translation, some terms are derived from the original work).

#### 3.3.12 De-stereotyping

Shi-Yan Chao claims that the development of Taiwan’s “*tongzhi*/queer movement” since the 1990s has made a “crucial intervention” to the “Chinese queer diasporic imaginary.” One of the impacts is the “deliberately joyful, positive portrayal of young gay men in pathbreaking documentary Boys for Beauty (美麗少年, dir. Mickey Chen, 1999),” which achieved unexpected commercial success and influenced a batch of queer films that followed. “In response to the ‘positivist politics’ of the ‘*tongzhi*/queer movement,’ the transformation of ‘this comic’ directly (though briefly) countered the ‘bleak tone’ that prevailed in the ‘Chinese queer diasporic imaginary’” (Chao, 2020a, p. 96, retain the original italics).

#### 3.3.13 Family

Shi-Yan Chao suggests that in Taiwanese *tongzhi*/queer films of the new millennium, present “the conspicuous absence of blood families, [……], involving young queer characters whose natal families have been rendered trivial, if not totally invisible.” Regarding the reasons for this phenomenon in the films, Shi-Yan Chao argues that it is due to the change in public attitudes toward homosexuality. As long as homosexuality does not enter their own families, they do not openly reject them (Chao, 2020a, p. 96).

#### 3.3.14 Diverse family formation/gender-diverse family

Shi-Yan Chao suggests that “the *tongzhi*/queer movement” intervening in “the Chinese queer diasporic imaginary” embodies a rethinking of the family. Among them, the film *Girlfriend Boyfriend* (aka, 女朋友○男朋友, dir. Yang Ya-che, 2012) presents the appearance of “diverse family formation” (Chao, 2020a, pp. 96, 97, retain the original italics). Zu-Xin Sun suggests that “in [Taiwan] queer films after 2012, the image of ‘diverse family formation’ has been continuously expressed and produced.” Zu-Xin Sun also cites the film *Girlfriend Boyfriend* as an example of a film that “opens up the visual imagination of ‘diverse family formation’ in the era of ‘marriage equality’” (Sun, 2023, p. 44, my translation, some terms are derived from the original work). Hsiu-Ping Tseng also mentions the possibility of a “gender-diverse family” when analysing the emotions of older gay and lesbian people in the film *Drifting Flowers*. “At the same time, the dichotomy of opposing tongzhi and heterosexual families is broken down and negotiated to a considerable degree” (Tseng, 2024, pp. 56, 57, my translation, some terms are derived from the original work).

#### 3.3.15 Gender performativity

Chun-Chi Wang claims that Judith Butler’s theory provides an excellent explanation for how Taiwanese tongzhi films address the issue of “gender deviance.” Taking Zero Chou’s three films as an example, she suggests that *Splendid Afloat* reveals Judith Butler’s pioneering viewpoint that the “how the stylisation of gender, governed by a rigid system, is naturalised” [Butler, 1999, cited in Wang (2020, p. 313)]. *Spider Lily* (aka, 刺青, dir. Zero Chou, 2006) and *Drifting Flowers* once again demonstrate “the fluidity across any widely recognised gender spectrum” (Wang, 2020, p. 313). Chun-Chi Wang argues that regardless of the biological sex, sexuality and gender can freely transform within the gender spectrum through the “stylisation of the body” (Wang, 2020, p. 313).

#### 3.3.16 Familial performativity

Shi-Yan Chao introduces the concept of “*familial performativity*” in his study of “the Chinese queer diasporic imaginary.” Shi-Yan Chao uses Tsai Ming-liang’s Taipei Trilogy- *Rebels of the Neon God* (aka, 青少年哪吒, dir. Tsai Ming-liang, 1992), *Vive L’amour* (aka, 愛情萬歲, dir. Tsai Ming-liang, 1994), and *The River* (aka, 河流, dir. Tsai Ming-liang, 1996) as examples, illustrating that “*familial performativity*” echoes Judith Butler’s notion of “gender performativity.” Based on the obligatory repetition of socially entrenched conventions about the “patrilineal family” while providing “the patrilineal family’s sense of originality and realness,” “*familial performativity*” is essential in the formation of the family subject in Chinese society as a whole, and because it excludes unqualified subjects, such as “homosexual individuals,” it is essential for the “figuration of the Chinese queer diasporic imaginary” (Chao, 2020a, p. 89, retain the original italics).

#### 3.3.17 Queer sphere

Ju-Ting Hsu argues that “the images of the new generation of female directors [in Taiwan] offer an imaginary ‘queer sphere, Utopia,’ the ‘queer sphere’ that is an appropriation of atypical modes of everyday life and an alternative space that subverts social norms” (Hsu, 2021, p. 77, my translation, some terms are derived from the original work). Based on interviews with director Tsai Ming-liang and research into his work, Nicholas de Villiers uses “queer film theory and approaches to queer diaspora, queer regionalism, and queer phenomenology” to explore “Tsai’s queering of space” (De Villiers, 2022).

#### 3.3.18 Queer gaze

Ju-Ting Hsu proposes to extend from “male gaze” and try to discuss it with “queer gaze.” “In the current gender culture that has moved toward fluidity and diversity, queer gaze is bound to be a diverse and fluid perspective.” However, the author did not expand on the discussion, just merely presented the idea and left it as a direction for future discussion (Hsu, 2021, p. 78, my translation, some terms are derived from the original work). Zu-Xin Sun argues that between 2002 and 2011, some gay/lesbian film creators chose to “actively expose the secret view in the closet, stimulating prying eyes and dissolving the power structure of the gaze.*”* In doing so, *“*brought about the queer impact on heteronormative censorship. [……] It resists the inroads of power in a way that dissolves voyeuristic expectations” (Sun, 2023, pp. 42, 43, my translation).

In the gay and lesbian (queer) gender power dimension of Taiwanese films, it involves an increasingly diverse range of code names. However, it is still relatively easy to identify some of the more significant code names in the study of Taiwanese tongzhi films, with *identity* receiving more attention before the 2010s, and *diverse family formation/gender-diverse family* becoming a hot topic of discussion following the legislative confirmation of same-sex marriage in Taiwan in 2019. What runs throughout is the expression of *indigenous* experience and the use of *narrative homonormativity* in Taiwanese tongzhi films.

Certainly, some problems have emerged. Gender power research on queer ethnic minorities and queer immigrants is not sufficient. Disabling queer gender power research expands the breadth of gender studies, and although it does not result in a wide-ranging discussion, it provides a direction for subsequent research.

As some scholars have highlighted, although the legalisation of same-sex marriage in Taiwan has put it at the forefront of Asia in terms of gender affirmation, it still faces many problems (Jhang, 2020; Wu, 2021). This seems to form an interesting intertext with the academic discussion of gay and lesbian (queer) power in Taiwanese cinema. In conducting the literature review, we found that most studies focused on the works of two tongzhi film directors, Zero Chou and Tsai Ming-liang, and concentrated on their works before the 2010s. However, there is a noticeable lack of attention and discussion regarding queer film works from the 2010s and 2020s. Therefore, at this point, both queer power in Taiwan and queer power in film still need more attention and discussion.

### 3.4 Phase 4. *Taiwan film chronology*

In analysing the literature, it can be seen that the entire sample of films selected for the literature focuses on the period after Taiwan’s restoration, from the 1950s to the 2020s. It should be noted, however, that although the number of Taiwanese films was limited during the colonial period, “tropes” with central plots revolving around female characters already existed (Wang, 2020, p. 303). Clarifying the chronological staging of Taiwanese films helps to understand the development of gender power dynamics more clearly in the context of Taiwan’s social and historical background, and at the same time clarifies the correlation between the three dimensions of men, women, and gay and lesbian (queer), the code names, and the chronology of the films. Based on the combing of related literature, Table 1 below shows the more common Taiwanese film staging.

**Table 1:** Taiwan film chronology.

Time division	Sign
1950s-1960s, mainly focuseded on Taiwanese-language film melodrama and spy films, and healthy realism films (Wang, 2020, pp. 303, 304; Shih, 2020, p. 115), and Qiong Yao films of the 1960s (Zhou, 2023, p. 367).	T1
The 1970s-1981, genre films period, mainly focused on Qiong Yao films of the 1970s (Wang, 2020, p. 305; Zhou, 2024, p. 93), and social realist film/Taiwan Pulp (including woman’s revenge film/rape-revenge films) and gangster film (sub-genres of crime films) (Wang, 2020, p. 306; Cho, 2024, p. 258; Chiang, 2022, p. 23, 2024, p. 23).	T2
1982–1987, Taiwan New Cinema (Pan, 2021).	T3
1988–2007, New New Wave/Post-New Cinema (Pan, 2021, p. 286; Pickowicz and Zhang, 2020, p. 5), meanwhile, focused on gangster film (sub-genres of crime films).	T4
2008, The Year of the Post-New Cinema (Pan, 2021; Fan, 2025, p. 768).	T5
2009–2019, Post-New Cinema (Pan, 2021; Fan, 2025, p. 768).	T6
2020s, 2020s Taiwan Contemporary Cinema.	T7

### 3.5 Phase 5. *Discussion*

Different gender groups are involved in different code names, and some code names are mentioned in the men, women, and gay and lesbian (queer) dimensions or in both dimensions at the same time. Through an extensive literature review, employing basic content analysis methodology, manually screen and identify sampling frames that align with the research theme and objectives, and then unite the data contained within these sampling frames. “Unitizing the data links the sampling and the coding processes”. Analysis sampling or *sampling units* [specific parts within a larger text that have been divided for easier management], identify relevant *recording units*, and assign associated code names [Weber, 1990, cited in Drisko and Maschi (2016: 41, retain the original italics)]. These code names are closely related to gender power dynamics within Taiwanese cinema. By analysing and discussing them, one can more clearly present the research dynamics concerning gender power in Taiwanese cinema over the past five years. In summary, thirty-six code names and three dimensions were identified, of which six code names were related to the men dimension, twelve code names were related to the women dimension, and eighteen code names were related to the gay and lesbian (queer) dimension. The frequency of each code name was recorded based on the literature review. “*Word frequencies* detail how often specified words are found in the text documents” (Drisko and Maschi, 2016: 50, retain the original italics). In this paper, “word” refers to a code name and denotes its frequency of occurrence within the sampling frame we have defined.

Each code name should be understood considering the corresponding year/era, because film is a cultural product affected by political, economic, and social development, so it is more accurate and persuasive to understand these code names in the context of the reality of a certain year/era. In the literature review above, three films deal with different code names, one of which is *Kano*, a film about Taiwan during the Japanese colonial period (1930s era); however, we use the year 2014 when the film was released as a reference standard, and put the code names dealt with in this film into the time dimension of the 2010s. Although the film reflects the story of Taiwan during the Japanese colonial period, the creators of the film reflect and present the story of 80 years ago in the 2010s; more or less, it incorporates the current thinking of the time. Therefore, we use the time of the film’s release as the basis for dividing the temporal dimension of the code names. Similarly, there is *Seediq Bale*. *Your Name Engraved Herein* is a 2020 film that spans the period from the post-martial-law era in Taiwan to the 21st century. We still use the time of film release as the basis for the division.

**Figure 2:**
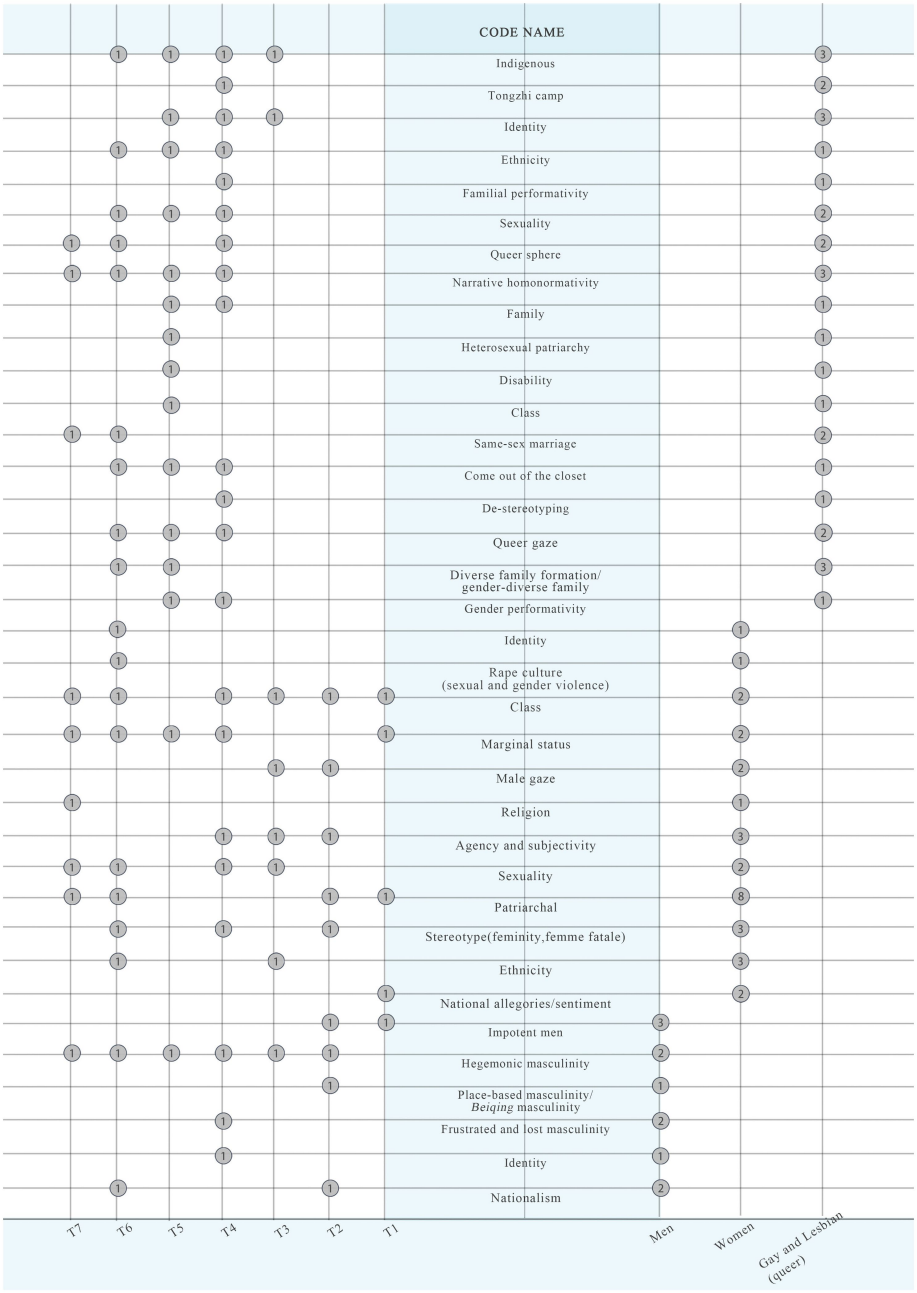
Bubble chart.

This paper adopts a bubble chart (Figure 2) to visualise the dynamics of gender power research in Taiwanese film in the past five years. The left side of the chart lists seven eras of Taiwanese cinema (refer to Table 1), the right side lists three dimensions of gender power, men, women, and gay and lesbian (queer), respectively, and the vertical axis lists the code names of each dimension. The data in the bubbles represent the frequency weights between the code names and the dimensions, and the corresponding year/era. Figure 2 shows that the *identity* appears in the men, women, and gay and lesbian (queer) dimensions. However, the focus of *identity* in the three dimensions is different, and relates to different eras; they all point to the dilemmas of individual or group identity under the pressure of the social environment. *Nationalism* is mentioned in both the men and women dimensions, suggesting that both males and females acted as symbols of nationalism in the post-war period. Four groups of code names emerged for the women and gay and lesbian (queer) dimensions, *ethnicity*, *class*, *patriarchy*, and *sexuality*, suggesting that women and gay and lesbian (queer) people face the same or similar problems in their individual development and society. In particular, ethnic minorities, immigrants, disability, and sexuality are areas that have received more attention recently, expanding the breadth of gender issues that deserve more attention and discussion.

## 4 Conclusion

This paper provides a more detailed literature review of studies related to gender power in Taiwanese cinema from 2020 to 2024 through an extensive literature search and careful manual screening. Taking 1950s Taiwanese-language films to 2020s contemporary Taiwanese films as the timeframe, it is divided into three dimensions: men, women, and gay and lesbian (queer), concerning thirty-six code names. Through these code names and the years/eras of distribution, the focus of academic attention on the study of gender power in Taiwanese cinema can be presented more clearly and can form a certain guidance for future research.

Through the bubble chart, the visualisation effect of the literature review is presented. It not only breaks through the existing traditional method of reviewing the literature and tries a new method, but also facilitates researchers and related personnel to understand more intuitively the development of the study of gender power in Taiwanese cinema. This study fills a gap in the literature on gender power research in Taiwanese films and provides a text to be used as a reference for the visualisation of literature review methods for gender power research in Taiwan and other regions.

The development of Taiwanese cinema over the past 70 years has created a wealth of fruitful results, in terms of genres, themes, markets, aesthetics, and so on, all of which have traced a rich tapestry of Chinese-language cinema and continue to exert their energy. Entering the new century, especially the 2020s, with the further deepening of globalisation, the arrival of the post-epidemic era, and the rise of artificial intelligence, many new issues have presented themselves, and Taiwanese cinema has reflected them to a certain extent. However, in conducting the literature review, we found that research on contemporary Taiwanese cinema in the 2020s is limited. Studies on male power in cinema seem to form a clever intertext with before 1970s studies of men and masculinity, with Gottzén et al. (2020, p. 1) arguing that men’s position has been largely ignored and treated as an “*absent presence*,” where they are not examined as “*gendered beings*”. Indeed, men and masculinity have had their rich trajectory in Taiwan (Shiau, 2020; Peng, 2021; Yau, 2023; Cho, 2024), and the gaps in the study of men and masculinity in cinema are very much worthy of further attention. Studies on gender power in Taiwanese cinema, especially those concerning ethnicity, immigration, sexuality, and disability, are scarce. The existence of these gaps guides subsequent studies, and their refinement is necessary and meaningful.

Finally, one of the more thought-provoking phenomena is the near gap in research on gender power in the year of the Post-New Cinema. The men’s and women’s dimensions in the T5 timeline (2008, The Year of the Post-New Cinema) discuss the presentation of gender power in the gangster genre in the bubble chart (Figure 2), while the gay and lesbian (queer) dimension discusses gender power in the 2008 film *Drifting Flower* by director Zero Chou. Strictly speaking, the film that launched the Post-New Cinema era, *Cape No. 7*, including several widely mentioned films that appeared before and after it (Pan, 2021, pp. 284, 288–289; Fan, 2025, p. 768), is almost devoid of any relevant academic discussion of gender power. We may understand that there has been no relevant academic discussion in the last five years; however, does this mean that to this day, these films are no longer needed or necessary to continue to discuss from the point of view of gender power? This is something that deserves deeper reflection.

## Footnotes

2 The term “*tongzhi*” has been interpreted by several scholars. See Jhang (2020), Kong (2023), and Wang (2020).

3 Chinese queer diasporic imaginary: Shi-Yan Chao ﻿﻿introduces﻿ this term in the book *Queer Representations in Chinese-language Film and the Cultural Landscape* (2020), mainly to counter the racial/ethnic insensitivity and unthinking Euro-American centrism in the concept of “*queer diaspora*.” Particular prominence is given to the “*Chinese experience*,” emphasising “the haunting influence of such notions as filiality [xiao (hanyu pinyin-Chinese phonetic)], familial-home [jia (hanyu pinyin-Chinese phonetic)] and family-state [jia-guo (hanyu pinyin-Chinese phonetic)] that are particular to Chinese cultural settings,” moreover, the term he ﻿﻿proposes﻿ challenges the “homogenizing and nationalist tendencies” that have underpinned much of the “Chinese diaspora discourse” since the 1990s. Concerning “*imaginary*,” he ﻿﻿suggests﻿ that “as a collection or archive of media representation, both audiovisual and literary.” Further, he defined the scope of his research, mainly, “Taiwanese media representation of queer subjects, [……], closer to ideas of diasporizing the queer [with internal exile at its core]” (Chao, 2020a, pp. 43, 44, 45, 46).
